# An Older Patient with a Symptomatic Arachnoid Cyst in the Velum Interpositum: Considerations of Functional Neuroanatomy

**DOI:** 10.1055/a-2678-8527

**Published:** 2025-08-21

**Authors:** Shunsuke Fujitsuku, Sadahiro Nomura, Hirokazu Sadahiro, Masami Osaki, Hideyuki Ishihara

**Affiliations:** 1 Department of Neurosurgery, Yamaguchi University School of Medicine, Ube, Yamaguchi, Japan

**Keywords:** arachnoid cyst, neuroendoscopy, performance IQ, route-finding disorientation, velum interpositum, visual–spatial construction apraxia

## Abstract

We report a patient with an arachnoid cyst in the velum interpositum (VI) and discuss the mechanism of the symptoms based on functional neuroanatomy. A 68-year-old woman presented with difficulty in doing housekeeping and with route-finding disorientation in known locations. Her performance intelligence quotient (PIQ) score was 68, significantly lower than her verbal intelligence quotient (IQ) of 103. Significantly low scores were obtained for the picture arrangement, picture completion, and symbol search tasks (4, 1, and 5, respectively) in the PIQ subtests. Her copies of the interlocking pentagons and cube designs were distorted, indicating visual–spatial construction apraxia. However, verbal IQ, working memory, urination control, ideational and ideomotor function, and dressing were intact. Magnetic resonance imaging revealed a cystic enlargement of the VI. Neuroendoscopic cyst fenestration to the lateral ventricles contributed to a decrease in the volume of the cyst. Postoperatively, her PIQ improved to 94. Her scores on the picture arrangement, picture completion, and symbol search tests increased to 7, 7, and 11 points, respectively. The pentagons and cube designs were copied correctly. An arachnoid cyst in VI is known to present with cognitive dysfunction. In our patient, symptoms were limited to the constructional apraxia and route-finding disorientation owing to the disturbance in the biparietal connections and posterior cingulate gyrus, respectively. The intramantle pressure gradient created by the characteristic cone-shaped cyst may have caused the selective dysfunctions. Namely, the impairment in the deep parietal region was more severe than on the frontal lobes or superficial parietal lobes.

## Introduction

The velum interpositum (VI), contrary to its appearance of being located inside the brain, is interposed between the bilateral hemispheres and the diencephalon. The posterior choroidal arteries and choroid plexus enter from the galenic cistern into the VI, run anteriorly, and exit at the foramen of Monroe. Internal cerebral veins (ICVs) enter the VI at the foramen of Monroe, run posteriorly, exit to the galenic cistern, and join the vein of Galen.

Dilatation of the VI space is normally documented during the fetal period and has been defined as cavum veli interpositi (CVI). The CVI usually decreases in size after birth 1 ; however, rare cases with persistent or enlarged CVI require treatment. Two theories of the anatomical definition of CVI exist. Kier 2 indicated that the CVI lies within the double-layered tela choroidea, not superior to it; therefore, ICVs are within the CVI, not inferior to it. On the other hand, Chen et al. 3 indicated that the CVI is a true cistern above the third ventricle, and ICVs form parts of the inferolateral boundaries of the CVI, but are not within it. The debate ended with the classification of two forms of enlarged VI; namely, Kier's CVI relating to enlarged VI, and Chen's CVI relating to an arachnoid cyst in the VI. 4 The location of ICVs is the key condition driving differentiation. An enlargement of the CVI or arachnoid cyst is suggested to be caused by head trauma followed by arachnoid adhesion, creating a ball valve mechanism between the VI and the galenic cistern. 5

CVI and an arachnoid cyst in VI seldom present with increased intracranial pressure (ICP), 6 instead, developmental delay, 7 8 psychiatric symptoms, 8 9 and memory disturbance 4 have been reported. In contrast, cavum septum pellucidum and cavum vergae, which commonly present with headache, are not associated with behavioral disturbance and tend to be less frequent. 10 11

Herein, we report an older patient with an arachnoid cyst in VI, presenting with selected symptoms among the cognitive dysfunctions, namely, a normal verbal intelligence quotient (IQ) and decreased performance IQ (PIQ). Her symptoms were reversed by neuroendoscopic fenestration. The mechanism of the symptoms caused by the arachnoid cyst location and morphology is discussed, with consideration of functional neuroanatomy.

## Case Presentation

**Fig. 1:**
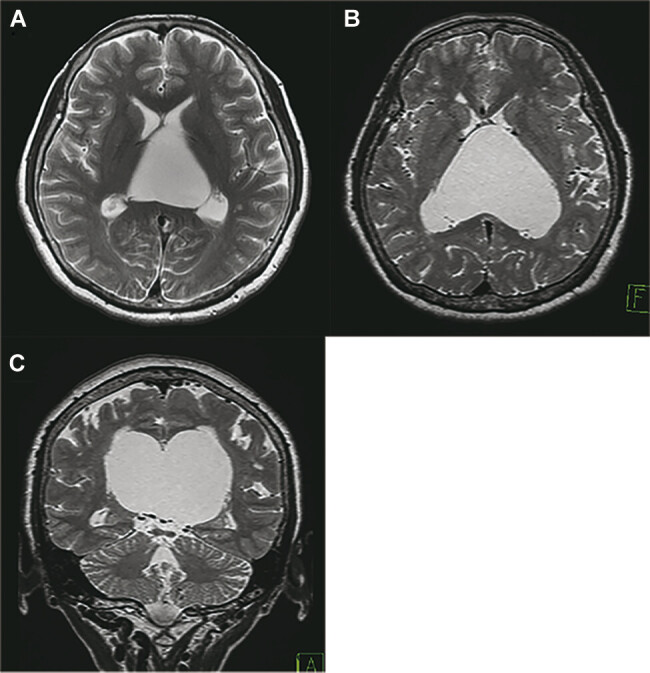
Serial head MRI before surgery. (A) Head MRI taken 8 years before admission. A cyst in the velum interpositum is revealed. (B, C) MRI taken before surgery. The cyst expands, and the lateral ventricle decreases in size. Internal cerebral veins (ICVs) are located at the bottom of the cyst. No tightness of sulci is observed. MRI, magnetic resonance imaging.

A 60-year-old woman, a right-handed homemaker and a high school graduate, who had no history of illness, underwent head magnetic resonance imaging (MRI) as part of a medical checkup, and dilatation of the VI was incidentally identified ( Fig. 1A ). She had no neurological symptoms at the time. Eight years later, she suffered from unstable gait, difficulty with housekeeping procedures, and getting lost when going out. She had no sign of increased ICP. However, MRI revealed an enlarged VI of 67 mm in longitudinal length, 77 mm in width, and 59 mm in vertical length. The corpus callosum and ICVs shifted upward and downward, respectively. These findings suggested a diagnosis of an arachnoid cyst in the VI rather than CVI. Her lateral ventricles were compressed laterally and had collapsed ( Fig. 1B, C ).

**Fig. 2:**
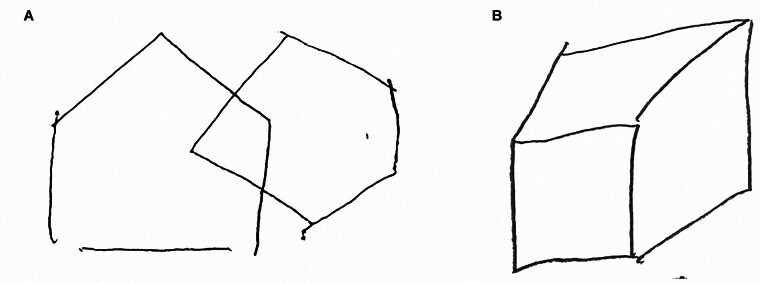
Preoperative drawing test of interlocking pentagons and cube. (A) The left equilateral pentagon transforms to a house base shape, and the right one rotates. (B) The cube distorts.

Preoperative cognitive tests were conducted, and she scored 28/30 points on the Mini-Mental State Examination (MMSE), 16/18 points on the Frontal Assessment Battery (FAB), and 86 on the full-scale IQ of the Wechsler Adult Intelligence Scale version III (WAIS-III; Table 1 ). Her PIQ was 68, significantly lower than her verbal IQ of 103. Among the subtests, significantly low scores were marked in picture arrangement, picture completion, and symbol search with 4, 1, and 5 points, respectively, and a normal score: 10 points. Her copies of the interlocking pentagons and cube designs were transformed, rotated, and distorted ( Fig. 2 ). She was able to use everyday tools, to gesture movements correctly, and she required no assistance with changing clothes, indicating no ideational apraxia, ideomotor apraxia, or dressing apraxia. Her gait was mildly unstable; however, it was fast enough at 11.2 seconds in the 3-meter Timed Up and Go test (3mTUG). Her neurological signs and symptoms were summarized as route-finding disorientation without landmark agnosia, and visual–spatial construction apraxia without visual–spatial agnosia.

**Table 1:** Scores of pre- and postoperative cognitive tests

		Age-matched average	Preop. score	Postop. score
WAIS-III				
Index scores	Full-scale IQ	100	86	100
	Verbal IQ	100	103	104
	Performance IQ	100	68	94
	Verbal comprehension	100	104	107
	Perceptual organization	100	72	95
	Working memory	100	107	103
	Processing speed	100	78	94
Verbal subtest	Vocabulary	10	10	10
	Similarities	10	9	11
	Information	10	13	13
	Comprehension	10	8	10
	Digit span	10	11	11
	Letter-number sequencing	10	12	9
	Arithmetic	10	11	12
Performance subtest	Picture arrangement	10	4	7
	Picture completion	10	1	7
	Block design	10	7	9
	Matrix reasoning	10	9	12
	Symbol search	10	5	11
	Digit symbol	10	7	7
MMSE		28 a	28	29
FAB		17.1 a	16	16

Abbreviations: FAB, Frontal Assessment Battery; IQ, intelligence quotient; MMSE, Mini-Mental State Examination; WAIS-III, Wechsler Adult Intelligence Scale Version III.

a Indicate the scores of the age and education-matched average. 13 14

## Surgical Findings

**Fig. 3:**
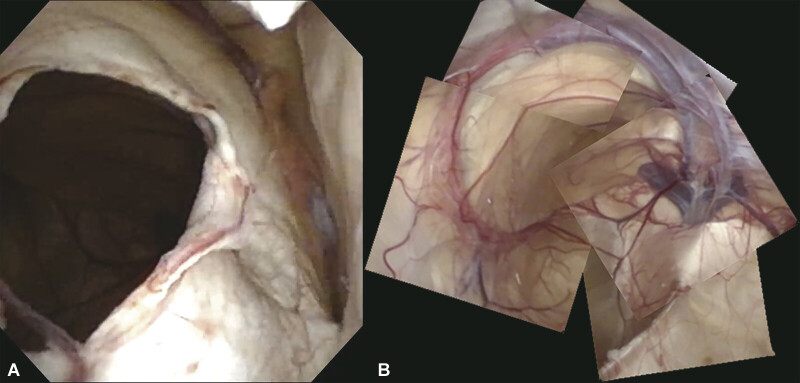
Endoscopic views in the lateral ventricle and the cyst. (A) Right anterior horn of the lateral ventricle. The septum pellucidum is fenestrated together with the cyst wall. The choroid plexus is seen on the right side. (B) Panorama picture reconstructed from the endoscopic photos. ICVs seen on the right side entering the vein of Galen are confirmed to be located at the bottom of the cyst. The choroid plexus of the left lateral ventricle is transparently seen through the cyst, and the ventricle wall is located on the left side. The crossing portion of the left ICV and the choroid plexus is the left foramen of Monroe. The upper side of the picture is the frontal side of the patient. ICV, internal cerebral vein.

Neuroendoscopic fenestration of the cyst was performed. A right lateral ventriculostomy was done through the frontal burr hole. The cerebrospinal fluid (CSF) opening pressure was 10 cm H _2_ O. Under observation with Videoscope® (Olympus, Tokyo, Japan), the septum pellucidum and cyst wall were fenestrated together ( Fig. 3A ). The ICVs were visualized at the bottom of the cyst ( Fig. 3B ). Through the location of the ICVs, the presence of an arachnoid cyst in the VI was confirmed. The body of the right lateral ventricle was identified from the location of the choroid plexus seen through the cyst wall, and a second fenestration was created there. By carefully avoiding the damage on the left crus of the fornix, we observed the pulsation of the cyst wall on the galenic cistern; however, no slit valve was found.

## Postoperative Course

**Fig. 4:**
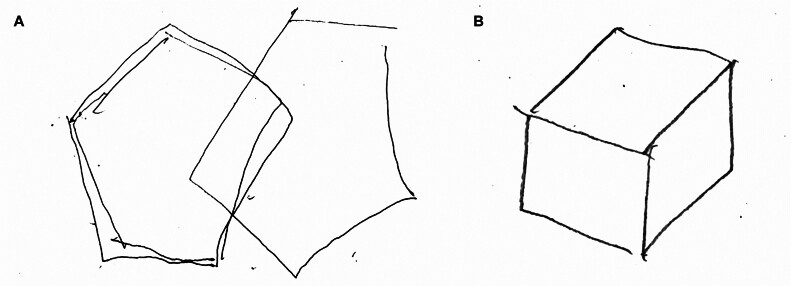
Postoperative drawing test of interlocking pentagons and a cube. (A) The transformation and rotation of the pentagons are corrected. (B) The distortion of the cube is corrected.

**Fig. 5:**
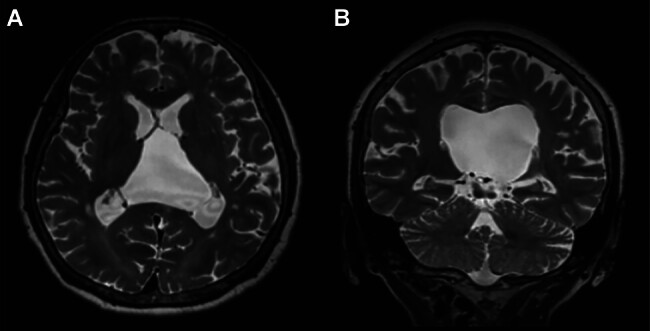
Postoperative MRI. (A, B) Head MRI taken 3 months after surgery. The cyst decreases, and the lateral ventricle increases in size. MRI, magnetic resonance imaging.

Three months postoperatively, she obtained a score of 29/30 points on the MMSE; 16/18 points on the FAB; and 100 for her full-scale IQ on the WAIS-III ( Table 1 ). Her PIQ had improved remarkably from 68 to 94. Among the subtests, scores on picture arrangement, picture completion, and symbol search increased from 4, 1, and 5 to 7, 7, and 11, respectively. She was able to copy the pentagons and cube designs correctly ( Fig. 4 ). Her gait was stable with 9.9 seconds in 3mTUG. MRI revealed that the cyst had decreased in size to 50 mm in longitudinal length, 60 mm in width, and 37 mm in vertical length ( Fig. 5 ). Although we had fenestrated the cyst wall bilaterally, only the right-sided stoma was patent.

## Discussion

A patient with an arachnoid cyst in VI was reported. The recovery with a decrease in cyst size indicated the symptoms were caused by compression and extension of the nerve fiber tracts in the white matter. 12 We herein discuss the mechanisms by which the frontal lobe dysfunction was mild and the parietal lobe functions were partly impaired.

### Frontal Lobe Functions

**Fig. 6:**
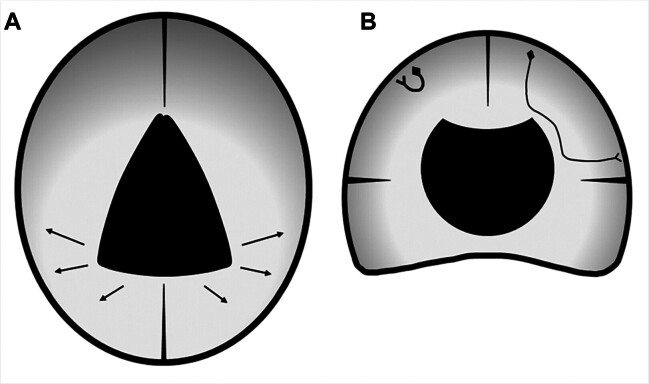
Schematic explanation of the mechanism of the topographically different influence from the cyst. (A) Axial view. The cone-shaped cyst (black triangle) compresses severely to the parietal lobes (arrows) and mildly to the frontal lobes. (B) Coronal view. A short nerve fiber located superficially (left side) is not damaged. A long fiber tract connecting the distant gyri (right side) and passing close to the cyst (black solid) is prone to compression. The grayscale of the brain indicates the parenchymal pressure gradient.

The patient presented with a mildly disturbed gait, small reductions in MMSE 13 and FAB, 14 and no disturbance in her verbal IQ, working memory, and urination control. As the VI is shaped like a cone, the apex, pointing to the foramen of Monroe, is thin compared with the wide bottom located between the crus of the fornix. Even when the arachnoid cyst in VI expanded, compression to the frontal lobes was still milder than that to the parietal lobes ( Fig. 6A ). Moreover, slit-like lateral ventricles due to compensated elimination of CSF in response to the expansion of the cyst avoided the frontal lobe compression. Therefore, emotion, motivation, urination control, and higher cerebral function controlled by the frontal lobes were within normal limits. The prefrontal cortico–ponto–cerebellar pathway for gait stabilization was not disturbed either; however, compression to the pyramidal tracts for legs located near the lateral ventricles in the posterior frontal lobes seemed responsible for her mildly disturbed gait.

### Parietal Lobe Functions

She exhibited low PIQ, especially in visual–spatial construction test, including the picture arrangement test, picture completion test, symbol search test, copies of the interlocking pentagons, and cube designs, but did not show other parietal dysfunction such as ideational apraxia, ideomotor apraxia, or dressing apraxia. The pressure gradient in the brain mantle of the parietal lobes ( Fig. 6B ) explains a suspected mechanism of the selective dysfunction in the patient. As the long fiber tracts connecting remote gyri run in the deep white matter, the cyst severely compressed and stretched the nerves. On the other hand, short fibers connecting within a gyrus or adjacent gyri run in the superficial portion of the brain, and thus, escape from compression. The visual–spatial construction function requires the bilateral parietal cortices and the corpus callosum connecting them. 15 16 17 18 Seydell-Greenwald et al. 17 reported the bilateral parietal activations in functional MRI under the visual spatial task. Compression of the corpus callosum caused her construction apraxia. Although she presented visual–spatial apraxia, she did not present visual–spatial agnosia or dressing apraxia. Those functions require only the right parietal lobe. She also retained the functions controlled by a single gyrus in the left parietal lobe, such as ideational function and ideomotor function.

### Topographical Disorientation

The patient suffered from determining the direction she should choose in known places where she recognized the streets and landmarks. Among topographical disorientation, landmark agnosia is a type of visual memory disturbance caused by a lesion in the occipitotemporal area, which the cyst of the patient did not affect. Her symptom is known as route-finding disorientation or heading disorientation. This function is controlled by the posterior cingulate cortex, 19 20 which was close to the cyst in the patient.

## Conclusion

A patient with an arachnoid cyst in VI presenting with constructional apraxia and route-finding disorientation was reported. Disturbance in the biparietal connections and the posterior cingulate gyrus caused the symptoms, respectively. The characteristic cone-shaped cyst affected the deep parietal region more severely than the frontal lobes or superficial parietal lobes.
